# Safety and effectiveness of ulotaront (SEP-363856) in schizophrenia: results of a 6-month, open-label extension study

**DOI:** 10.1038/s41537-021-00190-z

**Published:** 2021-12-09

**Authors:** Christoph U. Correll, Kenneth S. Koblan, Seth C. Hopkins, Yan Li, Heather Dworak, Robert Goldman, Antony Loebel

**Affiliations:** 1 Department of Psychiatry, The Zucker Hillside Hospital, Northwell Health, Glen Oaks, NY USA; 2 Department of Psychiatry and Molecular Medicine, Donald and Barbara Zucker School of Medicine at Hofstra/Northwell, Hempstead, NY USA; 3 Department of Child and Adolescent Psychiatry, Charité Universitätsmedizin, Berlin, Germany; 4 Sunovion Pharmaceuticals Inc., Marlborough, MA USA

**Keywords:** Schizophrenia, Schizophrenia

## Abstract

Ulotaront, a trace amine-associated receptor 1 (TAAR1) and serotonin 5-HT1A receptors agonist, has demonstrated efficacy in the treatment of patients with an acute exacerbation of schizophrenia in a 4-week, double-blind, placebo-controlled study. The aim of this 26-week open-label extension study was to evaluate the safety and effectiveness of ulotaront (25/50/75 mg/d) in patients who completed the initial 4-week study. Of the 193 4-week completers, 157 patients (81.3%) continued into the open-label extension study; 66.9% were completers. Among all extension phase patients, treatment with ulotaront was associated with minimal changes in body weight (mean [SD] change from double-blind baseline: −0.3 [3.7] kg), cholesterol (median change, −2.0 mg/dL), triglycerides (median, −5.0 mg/dL), and prolactin (female, median, −3.4 ng/mL; male, median, −2.7 ng/mL). Movement disorder scales showed no extrapyramidal effects. Twenty-six weeks of extension phase treatment was associated with a mean (95% CI) observed change from open-label baseline in the PANSS total score of −22.6 (−25.6, −19.6; effect size, 1.46), and a mean (95% CI) change in the CGI-Severity score of −1.0 (−1.2, −0.8; effect size, 1.07). Long-term treatment with the TAAR1 agonist ulotaront, in the daily dose range of 25–75 mg, was characterized by a relatively high completion rate, an adverse event profile notable for the absence of extrapyramidal-related adverse effects, a low liability for adverse weight and metabolic effects, and no effect on prolactin levels. Additional studies are needed to further confirm the long-term efficacy and safety of ulotaront.

## Introduction

Ulotaront (SEP-363856), one of the first of a new class of CNS-active compounds, is an agonist at trace amine-associated receptor 1 (TAAR1) as well as serotonin 5-HT1A receptors. TAAR1 is a G-protein-coupled receptor expressed in cortical, limbic, and midbrain monoaminergic regions that modulate dopaminergic, serotonergic, and glutamatergic activity^1–6^. In contrast to first- and second-generation antipsychotics, the efficacy of ulotaront is not mediated by blockade of D2 or 5-HT2A receptors^7^. Ulotaront has demonstrated positive effects in rodent models assessing endophenotypes of schizophrenia, including phencyclidine (PCP)-induced hyperactivity, prepulse inhibition, and PCP-induced deficits in social interaction and cognition^7,8^. In addition, ulotaront has also been shown to reduce ketamine-induced increases in striatal dopamine synthesis capacity in mice, suggesting the potential to modulate presynaptic dopamine dysfunction observed in schizophrenia patients^9^.

Suppression of rapid eye movement sleep has also been reported after single doses of ulotaront in both rats and humans and was utilized as a translational pharmacodynamic measure to guide dose selection in subsequent clinical trials in schizophrenia patients^10^.

In a prior 4-week, randomized, double-blind, placebo-controlled clinical trial, ulotaront, in doses of 50 or 75 mg, demonstrated significant efficacy in the short-term treatment of adults with an acute exacerbation of schizophrenia^11^. The study found ulotaront to have a safety and tolerability profile that appeared to differ from the profile exhibited by antipsychotic agents acting via D2/5-HT2A receptor binding mechanism described in the literature, including the absence of clinically meaningful effects on prolactin, extrapyramidal symptom (EPS) related adverse effects, and low liability for adverse weight and metabolic effects. While this is placebo-controlled trial lacked an active (D2 antagonist) comparator, the absence of ESP-related events and the absence of prolactin effects were notable.

We report here the results of the 26-week, open-label extension phase study (ClinicalTrials.gov Identifier: NCT02970929) for patients who completed the initial 4-week study^11^. This open-label study was designed to evaluate the long-term safety, tolerability, and effectiveness of ulotaront in patients with schizophrenia.

## Results

**Fig. 1:**
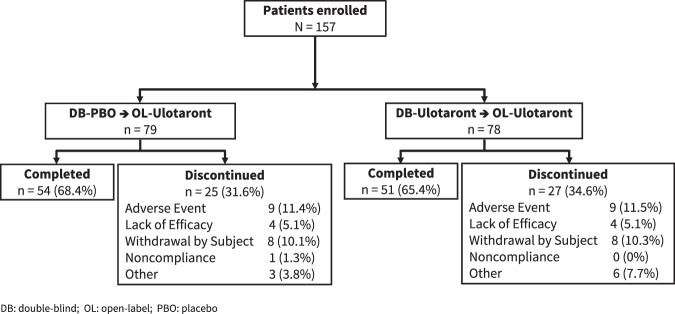
Patient disposition. Completion rates and categorization of reasons for discontinuation during 26 weeks of open-label treatment with ulotaront.

A total of 193 patients completed the 4-week, double-blind, placebo-controlled trial, of whom 157 patients (81.3%) provided informed consent and continued into the open-label extension study, including 78 patients initially randomized to double-blind ulotaront (and who continued on ulotaront in the open-label extension study) and 79 patients randomized to double-blind placebo (who were switched to ulotaront at entry into the open-label extension study; Fig. 1). Switching was accomplished while maintaining the double-blind of the initial 4-week trial. One patient discontinued from the extension study without receiving a dose of study medication and was excluded from all safety and efficacy analyses.

**Fig. 2:**
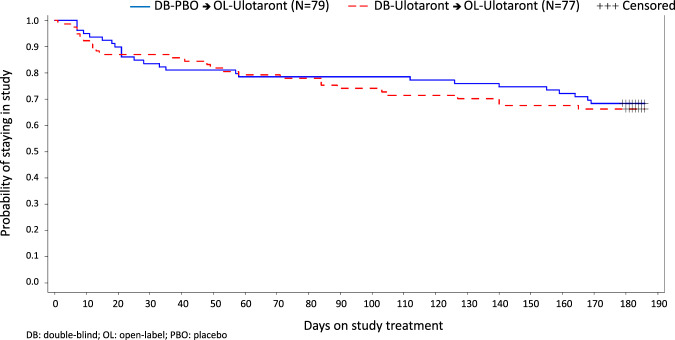
Kaplan–Meier estimate. Comparison of the probability of staying in the study during 26 weeks of open-label treatment for the two ulotaront subgroups.

Demographic and clinical characteristics at the extension phase baseline are summarized in Table 1. A total of 105 patients (66.9%) completed 26 weeks of open-label treatment. The Kaplan–Meier estimate of median time to discontinuation from open-label treatment is >26 weeks (Fig. 2). For the combined sample, reasons for premature discontinuation were adverse events (*n* = 18; 11.5%; 16 due to worsening of schizophrenia or psychosis), lack of efficacy (*n* = 8; 5.1%), patient withdrawal (*n* = 16; 10.2%), and miscellaneous other reasons (*n* = 10; 6.4%; Fig. 1).

**Table 1:** Characteristics of patients at open-label baseline (safety population).

Characteristic	DB-placebo → OL-ulotaront (*N* = 79)	DB-ulotaront → OL-ulotaront (*N* = 77)^b^
Male, *n* (%)	50 (63.3)	52 (67.5)
Age^a^, years, mean (SD)	30.2 (6.4)	30.2 (5.7)
Race, *n* (%)		
White	73 (92.4)	63 (81.8)
Black	5 (6.3)	10 (13.0)
Other	1 (1.3)	4 (5.2)
Hispanic, *n* (%)	4 (5.1)	1 (1.3)
Body mass index, kg/m^2^, mean (SD)	24.3 (3.2)	25.9 (4.3)
Time since initial onset of schizophrenia^a^, years, mean (SD)	4.3 (4.0)	5.2 (4.6)
Prior psychiatric hospitalizations^a^		
Mean (SD)	1.2 (0.8)	1.3 (0.7)
None, *n* (%)	18 (22.8)	11 (14.3)

*DB* double-blind, *OL* open-label.

^a^Age, time since initial onset of schizophrenia, and number of prior psychiatric hospitalizations are calculated at entry into the initial double-blind study.

^b^One patient discontinued without receiving a dose of study medication and was excluded from all safety and efficacy analyses.

Modal daily doses of ulotaront utilized during extension phase treatment were 25 mg/d (1.9% of patients), 50 mg/d (42.9%), and 75 mg/d (54.5%). Most patients (91.7%) met medication adherence criteria, based on pill counts and defined as taking 75–125% of prescribed doses during the study. In the safety population, the most frequently reported concomitant medications were anxiolytics (15.4%), hypnotics/sedatives (7.1%), and analgesics/antipyretics (7.1%).

### Effectiveness

Table 2 summarizes the effect of open-label extension phase treatment for the combined patient sample, and separately for double-blind ulotaront and double-blind placebo patients. On the Positive and Negative Syndrome Scale (PANSS) total score, mean (95% CI) observed change from open-label baseline to week 26 for double-blind ulotaront, double-blind placebo, and combined patients, respectively, was −17.1 (−20.6, −13.6), −27.9 (−32.5, −23.4), and −22.6 (−25.6, −19.6) (Table 2).

**Table 2:** Mean (SD) change from double-blind and open-label baselines to week 26 in efficacy measures by double-blind treatment assignment.

Efficacy measure	*n*	DB baseline mean (SD)	*n*	OL baseline mean (SD)	*n*	Mean (SD) change from DB baseline (OC)	*n*	Mean (SD) change from OL baseline (OC)	95% CI (effect size) for change from OL baseline (OC)^a^	*n*	Mean (SD) change from OL baseline (LOCF)
*PANSS total score*											
All extension phase patients	156	101.5 (8.0)	156	83.1 (15.0)	104	−41.8 (14.0)	104	−22.6 (15.5)	−25.6, −19.6 (1.46)	155	−13.8 (21.6)
DB-PBO to ulotaront	79	100.4 (7.7)	79	86.3 (13.4)	53	−41.2 (14.6)	53	−27.9 (16.4)	−32.5, −23.4 (1.70)	78	−17.2 (24.7)
DB-ulotaront to ulotaront	77	102.6 (8.2)	77	80.0 (16.0)	51	−42.4 (13.4)	51	−17.1 (12.3)	−20.6, −13.6 (1.39)	77	−10.3 (17.3)
*PANSS positive symptom*											
All extension phase patients	156	25.7 (3.2)	156	19.8 (5.0)	104	−13.5 (4.7)	104	−7.3 (5.4)	−8.3, −6.2 (1.36)	155	−4.5 (7.0)
DB-PBO to ulotaront	79	25.5 (3.2)	79	20.7 (4.7)	53	−13.4 (4.8)	53	−8.7 (5.7)	−10.2, −7.1 (1.53)	78	−5.6 (7.6)
DB-ulotaront to ulotaront	77	25.9 (3.3)	77	18.9 (5.1)	51	−13.5 (4.8)	51	−5.8 (4.7)	−7.2, −4.5 (1.25)	77	−3.3 (6.3)
*PANSS negative symptom*											
All extension phase patients	156	25.4 (4.0)	156	22.3 (4.4)	104	−8.4 (4.5)	104	−5.2 (4.2)	−6.0, −4.4 (1.24)	155	−3.5 (4.9)
DB-PBO to ulotaront	79	25.5 (4.2)	79	23.1 (4.2)	53	−8.5 (5.0)	53	−6.4 (4.6)	−7.6, −5.1 (1.38)	78	−4.1 (5.8)
DB-ulotaront to ulotaront	77	25.2 (3.9)	77	21.4 (4.4)	51	−8.3 (4.0)	51	−4.0 (3.4)	−4.9, −3.1 (1.19)	77	−2.9 (3.7)
*PANSS general psychopathology*											
All extension phase patients	156	50.4 (5.0)	156	41.1 (7.9)	104	−19.9 (7.9)	104	−10.2 (8.3)	−11.8, −8.5 (1.22)	155	−5.8 (11.4)
DB-PBO to ulotaront	79	49.3 (5.0)	79	42.5 (7.0)	53	−19.2 (8.4)	53	−12.9 (9.1)	−15.4, −10.4 (1.43)	78	−7.5 (13.1)
DB-ulotaront to ulotaront	77	51.5 (4.8)	77	39.6 (8.6)	51	−20.7 (7.3)	51	−7.3 (6.4)	−9.1, −5.5 (1.14)	77	−4.1 (9.2)
*CGI-Severity score*											
All extension phase patients	156	5.0 (0.4)	156	4.0 (0.8)	104	−2.0 (0.8)	104	−1.0 (0.9)	−1.2, −0.8 (1.07)	155	−0.6 (1.2)
DB-PBO to ulotaront	79	4.9 (0.4)	79	4.2 (0.7)	53	−2.1 (0.8)	53	−1.4 (0.9)	−1.7, −1.1 (1.51)	78	−0.9 (1.3)
DB-ulotaront to ulotaront	77	5.0 (0.4)	77	3.8 (0.9)	51	−1.8 (0.8)	51	−0.5 (0.7)	−0.7, −0.4 (0.82)	77	−0.3 (0.9)
*BNSS total score*											
All extension phase patients	148	38.4 (11.9)	150	33.0 (11.4)	96	−16.8 (12.4)	100	−11.3 (9.7)	−13.2, −9.3 (1.16)	149	−8.0 (11.2)
DB-PBO to ulotaront	76	38.3 (12.8)	77	34.6 (11.4)	50	−17.8 (13.5)	51	−14.4 (9.8)	−17.2, −11.7 (1.47)	76	−9.6 (12.7)
DB-ulotaront to ulotaront	72	38.5 (11.0)	73	31.4 (11.3)	46	−15.7 (11.2)	49	−8.0 (8.5)	−10.4, −5.6 (0.94)	73	−6.3 (9.1)
*UPSM PANSS NAA*											
All extension phase patients	156	2.6 (0.7)	156	2.3 (0.7)	104	−0.7 (0.8)	104	−0.4 (0.7)	−0.6, −0.3 (0.63)	155	−0.3 (0.7)
DB-PBO to ulotaront	79	2.6 (0.8)	79	2.4 (0.7)	53	−0.8 (0.9)	53	−0.6 (0.8)	−0.8, −0.4 (0.77)	78	−0.4 (0.8)
DB-ulotaront to ulotaront	77	2.5 (0.6)	77	2.2 (0.7)	51	−0.7 (0.7)	51	−0.3 (0.6)	−0.5, −0.1 (0.47)	77	−0.2 (0.6)
*UPSM PANSS NDE*											
All extension phase patients	156	2.1 (0.8)	156	1.9 (0.7)	104	−0.7 (0.8)	104	−0.5 (0.7)	−0.6, −0.3 (0.66)	155	−0.3 (0.7)
DB-PBO to ulotaront	79	2.1 (0.8)	79	2.0 (0.6)	53	−0.7 (0.9)	53	−0.6 (0.8)	−0.8, −0.4 (0.79)	78	−0.4 (0.8)
DB-ulotaront to ulotaront	77	2.2 (0.7)	77	1.9 (0.7)	51	−0.7 (0.7)	51	−0.3 (0.6)	−0.5, −0.1 (0.54)	77	−0.3 (0.7)
*MADRS total score*											
All extension phase patients	156	12.6 (7.2)	156	9.2 (6.3)	104	−8.1 (6.4)	104	−4.5 (5.3)	−5.6, −3.5 (0.86)	155	−2.2 (7.3)
DB-PBO to ulotaront	79	12.5 (7.3)	79	9.6 (6.2)	53	−8.2 (6.9)	53	−5.5 (6.1)	−7.2, −3.8 (0.90)	78	−2.9 (8.7)
DB-ulotaront to ulotaront	77	12.8 (7.2)	77	8.7 (6.5)	51	−8.0 (5.9)	51	−3.5 (4.0)	−4.6, −2.4 (0.87)	77	−1.5 (5.6)
*UPSA-B total score*											
All extension phase patients			156	75.9 (16.5)			113	+6.8 (11.5)	4.6, 8.9 (0.59)	134	+6.2 (11.6)
DB-PBO to ulotaront			79	75.8 (16.2)			58	+8.6 (13.2)	5.1, 12.0 (0.65)	67	+8.3 (12.8)
DB-ulotaront to ulotaront			77	76.0 (16.9)			55	+4.9 (9.1)	2.4, 7.3 (0.53)	67	+4.1 (9.9)

*DB* double-blind, *OL* open-label, *OC* observed case, *LOCF* last observation carried forward, *PBO* placebo, *CI* confidence interval, *SD* standard deviation, *PANSS* Positive and Negative Syndrome Scale, *CGI* clinical global impression, *BNSS* Brief Negative Symptom Scale, *UPSA-B* UCSD Performance-Based Skills Assessment-Brief Version, *MADRS* Montgomery–Åsberg Depression Rating Scale, *UPSM* uncorrelated PANSS score matrix, *NAA* negative apathy/avolition factor, *NDD* negative-deficit of expression factor.

^a^95% Confidence interval and within-group effect size shown for change from open-label baseline to week 26.

On the Clinical Global Impression, Severity (CGI-S) score, mean (95% CI) observed change from open-label baseline to week 26 was −0.5 (−0.7, −0.4) for double-blind ulotaront patients and −1.4 (−1.7, −1.1) for double-blind placebo patients (Table 2). A similar pattern of mean changes in symptom severity measures, from open-label baseline to week 26, was evident in double-blind ulotaront and double-blind placebo patients, respectively, for the PANSS positive subscale (−5.8 and −8.7), the PANSS negative subscale (−4.0 and −6.4), the PANSS general psychopathology subscale (−7.3 and −12.9), Brief Negative Symptom Scale (BNSS) total score (−8.0 and −14.4), uncorrelated PANSS score matrix (UPSM) Negative Apathy/Avolition Factor (NAA) score (−0.3 and −0.6), and the UPSM Negative-Deficit of Expression Factor (NDE) score (−0.3 and −0.6).

**Fig. 3:**
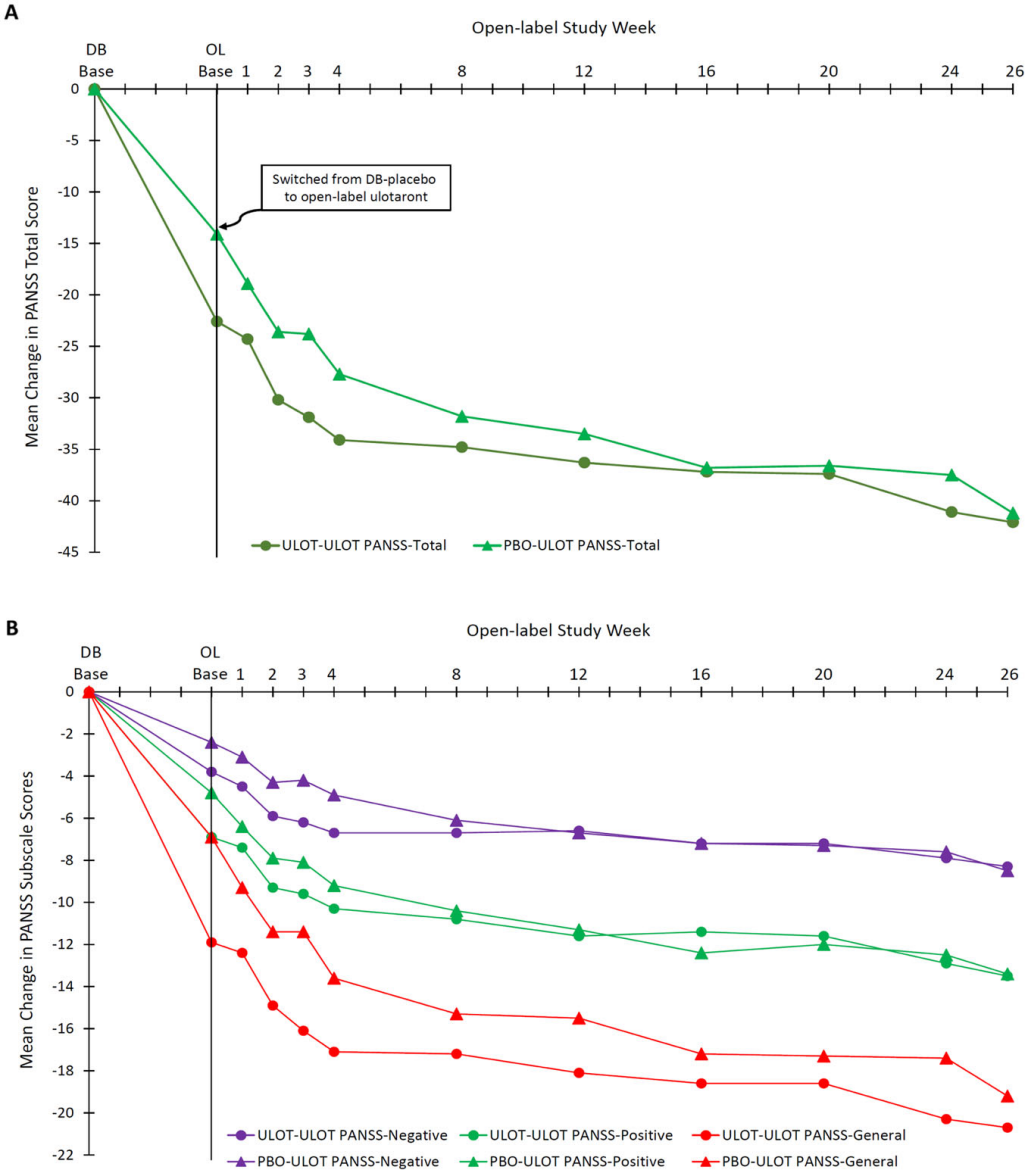
Mean change from double-blind baseline during 4 weeks of double-blind treatment with ulotaront or placebo and 26 weeks of open-label treatment with ulotaront. **A** Change in PANSS total score: observed case analysis. **B** Change in PANSS subscale scores: observed case analysis.

For double-blind ulotaront patients, both the PANSS total (Table 2 and Fig. 3A) and subscale scores (Table 2 and Fig. 3B) showed continued improvement during open-label treatment. Mean change from double-blind baseline to open-label baseline in PANSS total score was −22.6; and mean changes from double-blind baseline to open-label weeks 4, 12, 20 and 26 were −34.1, −36.3, −37.4, and −42.4, respectively.

Treatment response rates (≥30% reduction in PANSS total score from double-blind baseline) for double-blind ulotaront and double-blind placebo patients, respectively, were 94.1% and 92.5% at week 26 (observed case), and 74.0% and 73.1% at LOCF-endpoint; and response rates (≥50% reduction in PANSS) were 76.5% and 67.9% at week 26 (observed case) and 54.5% and 48.7% at LOCF-endpoint.

Depressive symptoms, measured by the Montgomery–Åsberg Depression Rating Scale (MADRS) total score, were mostly in the mild-to-moderate severity range at double-blind baseline (double-blind baseline mean for all extension phase patients, 12.6). The mean observed change from open-label baseline to week 26 for double-blind ulotaront and double-blind placebo patients, respectively, was −3.5 and −5.5 (Table 2). Observed improvement at week 26 was also evident on the University of California, San Diego, Performance-Based Skills Assessment-Brief version (UPSA-B) for double-blind ulotaront and double-blind placebo patients, respectively: mean change from open-label baseline +4.9 and +8.6, with a mean UPSA-B total score of 84 at week 26 for all extension phase patients combined.

Among the 51 patients initially randomized to double-blind ulotaront, and who achieved a clinical response after receiving 4 weeks of treatment, the Kaplan–Meier estimate (95% CI) of the probability of relapse at the end of the open-label extension treatment period was 0.23 (0.13, 0.38).

### Safety

In the open-label extension study, the overall incidence of AEs was 56.4%; 4 AEs occurred with an incidence ≥5% among all extension phase patients: schizophrenia (12.2%), headache (11.5%), insomnia (8.3%), and anxiety (5.1%; Table 3). The majority of AEs were either mild or moderate in severity, with 5.1% of patients reporting a severe AE. The only severe AE reported by more than one patient was schizophrenia (*n* = 5; 3.2%). A small improvement in sleep quality, as measured by the Pittsburgh Sleep Quality Index (PSQI) global score, was observed at week 26 (mean [SD] change from open-label baseline −2.0 [3.0]).

**Table 3:** Incidence of treatment-emergent adverse events (safety population).

Safety parameter, *n* (%)	OL-ulotaront^a^ (*N* = 156)
Schizophrenia	19 (12.2)
Headache	18 (11.5)
Insomnia	13 (8.3)
Anxiety	8 (5.1)
Somnolence	7 (4.5)
Nasopharyngitis	7 (4.5)
Nausea	6 (3.8)
Irritability	5 (3.2)
Influenza	5 (3.2)
Weight decreased	5 (3.2)
Prolactin increased	4 (2.6)
Extrapyramidal adverse events, any	5 (3.2)
Parkinsonism	2 (1.3)
Dyskinesia	1 (0.6)
Tremor	1 (0.6)
Restlessness	1 (0.6)
At least one adverse event	88 (56.4)
Adverse events rated as “severe”	8 (5.1)

Individual adverse events with an incidence ≥2% are shown (except for extrapyramidal adverse events).

*OL* open-label.

^a^All extension phase patients.

The incidence of AEs associated with EPS was low (*n* = 5; 3.2%; Table 3). Movement disorder scales showed no clinically meaningful changes from open-label baseline to week 26 (observed case): mean (SD) changes in the Simpson–Angus Scale (SAS) mean score, Barnes Rating Scale for Drug-Induced Akathisia (BARS) total score, and the Abnormal Involuntary Movement Scale (AIMS) total score were −0.0 (0.1), −0.1 (0.2), and 0.0 (0.1), respectively.

There were no deaths during the study. Suicidal ideation, assessed by the Columbia–Suicide Severity Rating Scale (C-SSRS), occurred in 3 patients, one of whom also reported suicidal behavior (aborted attempt on day 23; an adverse event of suicidal ideation started on day 18, study drug was discontinued on day 19, and the event resolved on day 30). Fifteen patients experienced a serious adverse event (SAE) of whom 12 reported an SAE of schizophrenia/psychotic disorder, one patient reported suicidal ideation and acute psychosis, one patient reported depression, and one had a uterine hemorrhage (unrelated to study drug).

Up to 26 weeks of open-label treatment with ulotaront was associated with minimal changes in body weight (mean observed change from double-blind baseline to week 26, −0.3 kg) or body mass index (−0.1 kg/m^2^; Table 4). The percent of patients with ≥7% increase or decrease in weight from open-label baseline to the LOCF-endpoint was 1.3% and 5.2%, respectively. No noteworthy changes were observed in metabolic laboratory parameters, including total cholesterol (median change from double-blind baseline to week 26, −2.0 mg/dL), triglycerides (−5.0 mg/dL), glucose (+2.0 mg/dL), or HbA1c (0.0%; Table 4). Treatment with ulotaront had no clinically meaningful effect on serum prolactin levels in either female (median change at week 26, −3.4 ng/mL) or males (−2.7 ng/mL; Table 4).

**Table 4:** Double-blind baseline values and change at week 26 in weight, body mass index, metabolic parameters, and prolactin (safety population; observed case analysis).

Safety parameter	Double-blind baseline		Week 26	
	*N*	Ulotaront	*N*	Ulotaront
Weight, kg, mean (SD)	156	75.4 (13.9)	104	−0.3 (3.7)
Body mass index, kg/m^2^, mean (SD)	156	25.1 (3.9)	104	−0.1 (1.2)
Total cholesterol, mg/dL, median	156	174.5	111	−2.0
LDL cholesterol, mg/dL, median	156	101.5	111	−9.0
HDL cholesterol, mg/dL, median	156	48.0	111	0.0
Triglycerides, mg/dL, median	156	101.0	111	−5.0
Glucose, mg/dL, median	156	92.0	109	+2.0
HbA1c, %, median	155	5.2	109	0.0
Prolactin, ng/mL, median				
Female	54	16.1	39	−3.4
Male	102	11.6	73	−2.7

Lipid and glucose data are shown for total available patients at week 26; 96.4% (107/111) of lipid results were fasted at week 26, 96.3% (105/109) of glucose results were fasted at week 26.

Ulotaront data are shown for all extension phase patients; mean baseline and change values are shown for weight and BMI; median baseline and change values are shown for laboratory parameters.

*HDL* high-density lipoprotein, *LDL* low-density lipoprotein.

Few clinically meaningful changes in vital signs occurred during 26 weeks of extension phase treatment. Eight patients (5.2%) met predefined criteria for orthostatic hypotension (decreased systolic blood pressure by ≥20 mmHg or decreased diastolic blood pressure by ≥10 mmHg) and 4 patients (2.6%) met criteria for orthostatic tachycardia (increase in heart rate by ≥20 beats per minute and heart rate >100 beats per minute). No AEs related to orthostatic hypotension or orthostatic tachycardia were reported. Overall, mean changes from open-label baseline in electrocardiogram parameters were small and not clinically meaningful. No patient had a QTcF interval ≥480 msec at any time point during the 26-week extension phase; one patient had an increase in QTcF interval from open-label baseline by ≥60 msec.

## Discussion

We report here the results of a multi-regional, open-label study of the safety and effectiveness of 26 weeks of flexible-dose treatment with ulotaront (25, 50, or 75 mg/d) in adults with schizophrenia who had completed an initial 4-week, double-blind, placebo-controlled study^11^. Long-term treatment with ulotaront, a TAAR1 and 5-HT1A receptor agonist, was characterized by an adverse event profile consistent with its non-D2 receptor binding mechanism of action. Notably, ulotaront was not associated with clinically meaningful changes in prolactin levels or in movement disorder scales (SAS, BARS, AIMS); and the incidence of Parkinson-like symptoms and akathisia were low. In addition, treatment with ulotaront was not associated with clinically meaningful effects on weight or metabolic parameters or increase in QTc interval. Three serious adverse events were reported that were not related to the underlying diagnosis of schizophrenia (suicidal ideation, depression, uterine hemorrhage). Overall, adverse event rates were low, with few events (5.1%) rated as severe. These long-term findings extend the tolerability results of the initial 4-week, double-blind study in which ulotaront was found to have a low (<7%) incidence of individual adverse events (for events that occurred more frequently in the ulotaront group), with each event minimally different from placebo (NNH ≥ 40)^11^. Taken together, the safety findings from the prior short-term^11^ and current long-term extension study suggest that ulotaront represents a well-tolerated treatment option for patients with schizophrenia.

The study completion rate after 26 weeks of open-label treatment with ulotaront (67%) was relatively high compared to 26-week completion rates of 39–65% reported previously in other long-term studies of atypical antipsychotics^12–14^. Estimated discontinuation rates at 6 months (based on KM plots) from the Clinical Antipsychotic Trials of Intervention Effectiveness (CATIE) study^13^ provide a useful benchmark for comparison with the current results given the “real world” features of the study. Olanzapine had the highest 6-month completion rate (55%), while rates were notably lower for risperidone (43%), perphenazine (41%), quetiapine (39%), and ziprasidone (38%). Medication discontinuation during maintenance therapy with antipsychotics has been shown to significantly increase rates of schizophrenia relapse and rehospitalization^15,16^. It is possible that the overall tolerability and safety of ulotaront observed in the current long-term, open-label study contributed to the relatively low rates of discontinuation over the treatment period.

In the current study, treatment with ulotaront was associated with small reductions in weight, total and LDL cholesterol, and triglycerides, and a small increase in glucose but no change in glycosylated hemoglobin. The low propensity of ulotaront for treatment-related increases in weight and metabolic parameters is especially important given the need for long-term treatment in patients with schizophrenia. Patients with a diagnosis of schizophrenia are an at-risk population with a marked increase in cardiovascular mortality, and a life expectancy 15–20 years shorter than in the general population^17,18^. Maintenance therapy with selected atypical antipsychotics is associated with a significant increase in weight and metabolic risk factors^17,18^. Given these findings, the overall safety profile of ulotaront, including a lack of clinically meaningful metabolic effects and movement disorder symptoms, appears distinguished from currently available treatment options.

During the initial double-blind study, the magnitude of improvement in PANSS total score observed was comparable to the improvement reported for atypical antipsychotics acting via D2 receptor blockade^19–24^. During long-term treatment with ulotaront, continued improvement was observed across a broad array of schizophrenia symptom measures, including PANSS positive, negative, and general psychopathology subscales. Improvement in PANSS total score appeared to persist throughout the 26-week, open-label study including over the last 6 weeks of treatment. These results are consistent with pre-clinical findings indicating the potential for antipsychotic activity associated with ulotaront^7^, and suggest that the mechanism of action of ulotaront, involving TAAR1 agonism, represents a potentially effective approach to the treatment of patients with schizophrenia. Specifically, the results of this long-term study indicate that ulotaront treatment was associated with sustained improvement in psychotic symptoms in patients with schizophrenia.

During extension phase treatment ulotaront was associated with continued improvement in depressive symptoms as measured by the MADRS. Clinically significant depressive symptoms occur in approximately 25% of patients and represent an important treatment consideration since the presence of depressive symptoms is associated with poorer functioning, quality of life, and treatment adherence^25–27^. The mean MADRS score for all extension phase patients at double-blind baseline was 12.6, indicating mild-to-moderate levels of depressive symptomatology in most patients. By week 26, the mean MADRS score for all patients was <5, consistent with an absence of clinically meaningful depressive symptomatology.

Study limitations include an open-label, uncontrolled design for the extension study, and enrollment limited to patients ≤40 years at entry into the initial double-blind trial^11^. Furthermore, patients initially randomized to ulotaront in the double-blind portion of the study more likely continued in the open-label extension phase if they derived benefit from and tolerated ulotaront. However, 50.3% of the sample consisted of patients originally randomized to placebo who had an unknown response and tolerability to ulotaront, mitigating a potential selection bias that might otherwise have affected the results.

In conclusion, in this open-label extension study, ulotaront, one of the first of a new class of TAAR1 agonists, in the daily dose range of 25–75 mg, was generally safe, well-tolerated, and effective in the long-term treatment of patients with schizophrenia. Treatment with ulotaront over a 6-month study period was characterized by relatively high completion rates, an adverse event profile that was notable for the low rate of extrapyramidal-related adverse effects, a low liability for adverse weight, and metabolic effects, and no effect on prolactin levels. Additional studies are needed to further confirm the long-term efficacy and safety of ulotaront.

## Methods

This was a multi-regional, open-label extension study (consisting of 26 weeks of open-label treatment with ulotaront plus 1 week of follow-up off study drug) designed to evaluate the long-term safety, tolerability, and effectiveness of ulotaront for the treatment of adults with schizophrenia who completed the previously reported 4-week double-blind, placebo-controlled study (NCT # NCT02969382)^11^. Entry into the preceding acute study was limited to patients 18 to 40 years of age who met DSM-5 criteria for schizophrenia^28^ for at least 6 months with a Positive and Negative Syndrome Scale^29^ (PANSS) total score ≥80 (for more details see Koblan and colleagues^11^).

The study was conducted from January 2017 to January 2019 at 32 clinical sites in 5 countries (Hungary, Romania, Russia, Ukraine, and the United States). The study was approved by an Institutional Review Board/ethics committee at each investigational site and was conducted in accordance with the International Conference on Harmonisation Good Clinical Practices guidelines and with the ethical principles of the Declaration of Helsinki. After a full explanation of the study was provided, written informed consent was obtained from all patients.

Patients who met entry criteria were transitioned directly from the acute study, with the final week 4 visit of the acute study serving as the baseline visit of the current open-label study. Hospitalization was permitted during the first week of the current study, if deemed appropriate by the Investigator. To maintain the double-blind of the initial 4-week trial, patients enrolled in the current extension study received a starting ulotaront dose of 50 mg/d for 3 days, regardless of their initial double-blind treatment assignment (ulotaront or placebo). Beginning on day 4, up-titration to 75 mg/d was permitted but not required. From the week 1 visit, flexible doing from 25 to 75 mg/d was permitted. Changes in dose were made in increments/decrements of 25 mg.

Patients were assessed at weekly intervals for the first 4 weeks and then every 4 weeks thereafter up to week 24; a final end-of-treatment assessment was performed at week 26, and a follow-up visit at week 27. Safety assessments included monitoring for adverse events (AEs) and serious adverse events (SAEs), evaluation of vital signs and weight, body mass index, waist circumference, laboratory tests (including fasting lipid and glucose levels), and 12-lead electrocardiography. Extrapyramidal symptoms (EPS) were assessed by the Simpson–Angus Scale^30^ (SAS), the Barnes Rating Scale for Drug-Induced Akathisia^31^ (BARS), and the Abnormal Involuntary Movement Scale^32^ (AIMS). Suicidality was assessed by the Columbia–Suicide Severity Rating Scale^33^ (C-SSRS). Sleep quality was assessed by the Pittsburgh Sleep Quality Index^34^ (PSQI).

Effectiveness was assessed by the PANSS total and subscale scores, the Clinical Global Impression, Severity^32^ (CGI-S) scale, the Brief Negative Symptom Scale^35^ (BNSS), the Montgomery-Åsberg Depression Rating Scale (MADRS)^36^, and the University of California, San Diego, Performance-Based Skills Assessment-Brief version (UPSA-B)^37^. Effectiveness was also assessed using the uncorrelated PANSS score matrix (UPSM)-transformed PANSS factors which measure drug effects on clinical symptom domains of schizophrenia with greater specificity by correcting for correlated improvements among the individual PANSS items^38^.

Rate of relapse and time to relapse were evaluated during the 26-week open-label treatment period for patients who demonstrated a clinical response to 4 weeks of treatment with ulotaront in the double-blind phase. Clinical response was defined as ≥20% improvement in PANSS total score from double-blind baseline and CGI-S score ≤4. Relapse was defined as the earliest occurrence of any of the following: (1) ≥30% increase in PANSS total score from the PANSS score at the time of clinical response and a CGI-S score ≥ 3; (2) rehospitalization for worsening of psychosis; or (3) emergence of suicidality, homicidality, and/or risk of harm to self or others.

### Statistical methods

The safety population consisted of all patients who were enrolled and received at least one dose of ulotaront. The safety analysis was primary and included assessment of the incidence of AEs, SAEs, and AEs leading to study discontinuation, summarized descriptively in terms of incidence, event count, and severity. Clinical laboratory tests and vital signs, body weight, body mass index, waist circumference, and 12-lead electrocardiograms were calculated for change from double-blind and open-label baselines and are summarized descriptively. The frequency and severity of suicidal ideation and suicidal behavior using the C-SSRS were also provided.

Descriptive statistics were calculated for change in PANSS total and subscale scores, PANSS UPSM factor scores, CGI-S score, BNSS total score, MADRS total score, and UPSA-B total score, including means, standard deviations, 95% confidence intervals (CI), and within-group effect sizes (mean open-label baseline to week 26 change score divided by the standard deviation of the change). Kaplan–Meier estimates of the median time to discontinuation from the 26 weeks of open-label treatment were calculated for double-blind ulotaront patients and double-blind placebo patients. In the group of patients meeting clinical response criteria, Kaplan–Meier estimates of the probability of relapse were calculated for double-blind ulotaront patients at day 187 (counting from the clinical response), and for double-blind placebo patients at day 159 (counting from the clinical response).

### Reporting summary

Further information on research design is available in the Nature Research Reporting Summary linked to this article.

## Supplementary information

Reporting Summary

## Data Availability

Sunovion Pharmaceuticals Inc. is part of a clinical trial data-sharing consortium that facilitates access for qualified researchers to selected anonymized clinical trial data. For up-to-date information on data availability please visit https://www.clinicalstudydatarequest.com and click on Sunovion.
