# Supplementary material for: Safety and effectiveness of ulotaront (SEP-363856) in schizophrenia: results of a 6-month, open-label extension study
Source: NPJ Schizophr. 2021 Dec 9;7:63. doi: 10.1038/s41537-021-00190-z (PMC8660889; doi:10.1038/s41537-021-00190-z)
Supplement: Supplementary file 1 — Reporting Summary [file 41537_2021_190_MOESM1_ESM.pdf]

nature portfolio

Corresponding author(s): Christoph U. Correll, MD

Last updated by author(s): Sep 16, 2021

Reporting Summary

Nature Portfolio wishes to improve the reproducibility of the work that we publish. This form provides structure for consistency and transparency in reporting. For further information on Nature Portfolio policies, see our Editorial Policies and the Editorial Policy Checklist.

Statistics

For all statistical analyses, confirm that the following items are present in the figure legend, table legend, main text, or Methods section.

n/a	Confirmed	
☐	☒	The exact sample size (n) for each experimental group/condition, given as a discrete number and unit of measurement
☐	☒	A statement on whether measurements were taken from distinct samples or whether the same sample was measured repeatedly
☒	☐	The statistical test(s) used AND whether they are one- or two-sided<br>*Only common tests should be described solely by name; describe more complex techniques in the Methods section.*
☒	☐	A description of all covariates tested
☒	☐	A description of any assumptions or corrections, such as tests of normality and adjustment for multiple comparisons
☐	☒	A full description of the statistical parameters including central tendency (e.g. means) or other basic estimates (e.g. regression coefficient) AND variation (e.g. standard deviation) or associated estimates of uncertainty (e.g. confidence intervals)
☒	☐	For null hypothesis testing, the test statistic (e.g. F, t, r) with confidence intervals, effect sizes, degrees of freedom and P value noted<br>*Give P values as exact values whenever suitable.*
☒	☐	For Bayesian analysis, information on the choice of priors and Markov chain Monte Carlo settings
☒	☐	For hierarchical and complex designs, identification of the appropriate level for tests and full reporting of outcomes
☐	☒	Estimates of effect sizes (e.g. Cohen's d, Pearson's r), indicating how they were calculated

Our web collection on statistics for biologists contains articles on many of the points above.

Software and code

Policy information about availability of computer code

Data collection	The study used these systems for data collection: Electronic Data Capture (MediData RAVE); Laboratory Information Management System (LIMS); Core Lab Over-read; LIMS/ASCII; Interactive Response System; Bracket; CogState. A detailed description of the data collection systems can be found in study protocol, Section 16
Data analysis	All data analyses were conducted using SAS version 9.4

For manuscripts utilizing custom algorithms or software that are central to the research but not yet described in published literature, software must be made available to editors and reviewers. We strongly encourage code deposition in a community repository (e.g. GitHub). See the Nature Portfolio guidelines for submitting code & software for further information.

Data

Policy information about availability of data

All manuscripts must include a data availability statement. This statement should provide the following information, where applicable:

- Accession codes, unique identifiers, or web links for publicly available datasets
- A description of any restrictions on data availability
- For clinical datasets or third party data, please ensure that the statement adheres to our policy

Sunovion Pharmaceuticals Inc. is part of a clinical trial data sharing consortium that facilitates access for qualified researchers to selected anonymized clinical trial data. For up-to-date information on data availability please visit https://www.clinicalstudydatarequest.com and click on Sunovion.

Field-specific reporting

Please select the one below that is the best fit for your research. If you are not sure, read the appropriate sections before making your selection.

☒ Life sciences ☐ Behavioural & social sciences ☐ Ecological, evolutionary & environmental sciences

For a reference copy of the document with all sections, see nature.com/documents/nr-reporting-summary-flat.pdf

Life sciences study design

All studies must disclose on these points even when the disclosure is negative.

Sample size	The current study is a 6-month extension study in which all patients who completed an initial 4 weeks of double-blind treatment with ulotaront or placebo were given the opportunity to continue treatment with open-label ulotaront, or (if they were treated with double-blind placebo) switched to ulotaront. Therefore, sample size was determined by the number of 4-week study completers who provided consent to enter the current extension study.
Data exclusions	There were no data exclusions.
Replication	No specific measures were taken to verify the reproducibility of the experimental findings.
Randomization	This was not a randomized clinical trial. It was an open-label extension study with only one treatment group.
Blinding	This was not a blinded clinical trial. It was an open-label extension study with only one treatment group.

Reporting for specific materials, systems and methods

We require information from authors about some types of materials, experimental systems and methods used in many studies. Here, indicate whether each material, system or method listed is relevant to your study. If you are not sure if a list item applies to your research, read the appropriate section before selecting a response.

Materials & experimental systems

n/a	Involved in the study	
☒	☐	Antibodies
☒	☐	Eukaryotic cell lines
☒	☐	Palaeontology and archaeology
☒	☐	Animals and other organisms
☐	☒	Human research participants
☐	☒	Clinical data
☒	☐	Dual use research of concern

Methods

n/a	Involved in the study	
☒	☐	ChIP-seq
☒	☐	Flow cytometry
☒	☐	MRI-based neuroimaging

Human research participants

Policy information about studies involving human research participants

Population characteristics	The covariate-relevant population characteristics of the human research participants included the following: age, gender, race, ethnicity (Hispanic), physical characteristics (body mass index), psychiatric diagnosis and history (including time since initial onset of schizophrenia, history of prior psychiatric hospitalization, open-label baseline illness severity as measured by the Positive and Negative Syndrome Scale, Clinical Global Impression, Severity scale, the Brief Negative Symptom Scale, the Montgomery-Åsberg Depression Rating Scale, and the University of California, San Diego, Performance-Based Skills Assessment-Brief versions. These characteristics are summarized in the manuscript in Table 1 and Table 2. As noted in the first paragraph of the Methods section, all patients were required to be 18 to 40 years of age and to meet DSM-5 criteria for schizophrenia for at least 6 months with a Positive and Negative Syndrome Scale (PANSS) total score ≥80 at the time of entry into the initial double-bind 4-week study.
Recruitment	All participants in the current 6-month extension study had just completed an initial randomized, double-blind, 4-week ulotaront versus placebo treatment study. Enrollment in the current extension study was optional.
Ethics oversight	The study protocol and consent form were reviewed and approved by Ethics Committees at each of the investigational sites. The study was conducted in accordance with the International Conference on Harmonisation Good Clinical Practices guidelines and with the ethical principles of the Declaration of Helsinki.

Note that full information on the approval of the study protocol must also be provided in the manuscript.

Clinical data

Policy information about clinical studies

All manuscripts should comply with the ICMJE guidelines for publication of clinical research and a completed CONSORT checklist must be included with all submissions.

Clinical trial registration	ClinicalTrials.gov Identifier: NCT02970929
Study protocol	The Protocol for this study is available to qualified researchers from the corresponding author.
Data collection	Data was collected from participants while they were in the study by investigators and raters at each investigational site
Outcomes	The safety analysis was primary and included assessment of adverse events, serious adverse events, and adverse events leading to study discontinuation, summarized descriptively in terms of incidence, event count, and severity. Clinical laboratory tests and vital signs, body weight, body mass index, waist circumference, and 12-lead electrocardiograms were calculated for change from double-blind and open-label baselines and are summarized descriptively. The frequency and severity of suicidal ideation and suicidal behavior using the Columbia–Suicide Severity Rating Scale were also provided.<br>Descriptive statistics were calculated for change in Positive and Negative Syndrome Scale (PANSS) total and subscale scores, uncorrelated PANSS score matrix (UPSM) factor scores, the Clinical Global Impression Severity scale score, the Brief Negative Symptom Scale total score, the Montgomery-Åsberg Depression Rating Scale total score, and the University of California, San Diego, Performance-Based Skills Assessment-Brief version. Descriptive statistics included means, standard deviations, 95% confidence intervals (CI), and within-group effect sizes (mean open-label baseline to week 26 change scores divided by the standard deviation of the change). Kaplan-Meier estimates of the median time to discontinuation from the 26 weeks of open-label treatment were calculated for double-blind ulotaront patients and double-blind placebo patients. In the group of patients meeting clinical response criteria, Kaplan-Meier estimates of probability of relapse were calculated for double-blind ulotaront patients at Day 187 (counting from clinical response).
